# Relationship between Subjective Grip Strength and Physical Functioning among Community-Dwelling Older Women

**DOI:** 10.3390/geriatrics9030068

**Published:** 2024-05-26

**Authors:** Kohei Iwamoto, Yuki Kikuchi, Hideki Nakano, Tsuyoshi Katsurasako, Kohei Mori, Kayoko Shiraiwa, Jun Horie, Shin Murata

**Affiliations:** 1 Department of Physical Therapy, Faculty of Rehabilitation Science, Kobe International University, Kobe 658-0032, Japan; 2 Graduate School of Health Sciences, Kyoto Tachibana University, Kyoto 607-8175, Japan; 3 Department of Physical Therapy, Faculty of Health Sciences, Kyoto Tachibana University, Kyoto 607-8175, Japan; 4 Koka City Health and Welfare Department, Community Coexistence Promotion Division, Koka 528-8502, Japan; 5 Faculty of Allied Health Sciences, Kansai University of Welfare Sciences, Osaka 582-0026, Japan

**Keywords:** older adults, subjective motor ability, subjective grip strength, physical function, cognitive function

## Abstract

This study investigated the relationship between subjective grip strength and physical function in community-dwelling older women. Subjective grip strength was assessed using a questionnaire, and physical function and body composition were compared between groups with strong and weak subjective grip strength. Additionally, the two groups were compared in those with mild cognitive impairment (MCI) and those with normal cognitive function, respectively. The results showed significant differences in grip strength (*p* < 0.001), 30 s chair–stand (CS-30) test (*p* = 0.039), timed up-and-go (TUG) test (*p* = 0.027), maximal gait speed (*p* = 0.029), and skeletal muscle mass (*p* < 0.001). Older adults with normal cognitive function showed significant differences in grip strength (*p* < 0.001), quadriceps muscle strength (*p* < 0.009), one-leg standing time (*p* = 0.041), CS-30 (*p* = 0.002), TUG (*p* = 0.014), gait speed (*p* = 0.006), and skeletal muscle mass (*p* = 0.003). Older adults with low subjective grip strength had lower physical function and skeletal muscle mass. However, no items showed significant differences between groups among older adults with MCI. Thus, subjective grip strength is an indicator of an overall decline in physical function and a reduction in skeletal muscle mass in older adults, and cognitive function should be considered when assessing subjective grip strength in older adults.

## 1. Introduction

Population aging is a global phenomenon and problem common to many countries [1], not just developed ones. One of the problems associated with an aging society is an increase in social security costs. In Japan [2], which has the world’s highest aging rate as of 2021, social security expenditures are on the rise [3]. National healthcare expenditures are dominated by individuals aged ≥ 65 years, who account for 60.6% of total expenditures [4]. Given that the number of older adults is expected to continue to increase worldwide [3], the health promotion of older adults is an important issue.

Preventive interventions for frailty are effective in maintaining and improving physical function in older individuals [5,6]. However, with limited social resources, it is necessary to identify older adults with a high need for interventions and prioritize interventions in this population. Therefore, a simple screening method is required to determine the need for intervention.

Subjective indicators are simple and are thus useful for screening older adults. Previous studies have used subjective motor ability as an evaluation index. Subjective gait speed has been reported as an indicator of the risk of cardiovascular events [7,8]. In addition, subjective gait speed and cognitive function decline serve as predictors of dementia onset [9]. These reports suggest that subjective motor ability is a useful indicator of disease risk. Also, it is assessed using a single questionnaire [7,8,9] and thus can be performed in a short time with little burden on participants. Therefore, it is a suitable screening method for older adults.

Previous studies in young adults have examined the relationship between subjective motor ability and physical function using subjective gait speed and grip strength. The results showed that subjective grip strength was more strongly associated with physical function than subjective gait speed [10]. Therefore, this study focused on subjective grip strength. Because actual grip strength in older adults is related to lower limb muscle strength [11], gait speed [12,13], and balance ability [13], it is considered a suitable item for assessing subjective motor ability. Subjective grip strength can easily be assessed using a questionnaire [10]. Therefore, it is useful in situations where it is difficult to measure physical function, such as in large-scale health surveys, mailed questionnaires, and Internet-based health surveys. Furthermore, it can be used as a screening tool to determine the need for preventive interventions by assessing physical function.

This study aimed to identify the relationship between subjective grip strength and physical function in older adults. Previous studies have reported that predicted and measured physical function values dissociate in older adults with cognitive decline [14,15]. Therefore, this study examined the relationship between subjective grip strength and physical function while considering cognitive function assessed using the Mini-Mental State Examination (MMSE).

## 2. Materials and Methods

### 2.1. Subjects

**Figure 1:**
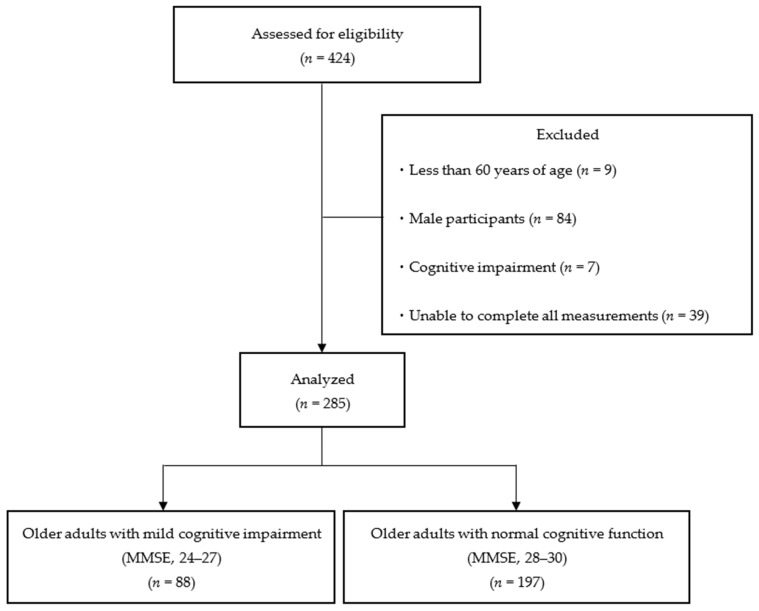
Flowchart of participation criteria. MMSE, Mini-Mental State Examination.

The study included 424 individuals (age: 76.8 ± 6.6 years) who participated in the physical fitness sessions conducted between September and October 2023. The participants were older adults who were independent in their daily lives and were able to come to the venue of the physical fitness test by themselves. The inclusion criteria were as follows: (1) age ≥ 65 years, (2) female sex, and (3) MMSE ≥ 24 points. The exclusion criteria were as follows: (1) age < 65 years, (2) male sex, (3) cognitive decline (MMSE score < 24 points), and (4) inability to perform all assessments. After excluding 139 participants, 285 participants were included in the analysis (Figure 1). The participants were informed of the purpose and content of the study in advance, and their consent was obtained before the measurements began. This study was approved by the Research Ethics Committee of Kyoto Tachibana University (approval number: 23-33) and conducted according to the guidelines of the Declaration of Helsinki.

### 2.2. Subjective Grip Strength 

Subjective grip strength was assessed using a questionnaire. Participants were asked the question “Compared with people of the same age and sex, do you think your grip is stronger?” and provided with four options: “Agree”, “Moderately Agree”, “Moderately Disagree”, and “Disagree”. Those who responded as “Agree” or “Moderately Agree” were classified as having a strong subjective grip strength, whereas those who responded as “Moderately Disagree” or “Disagree” were categorized as having weak subjective grip strength [10].

### 2.3. Cognitive Function

Cognitive function was assessed using the MMSE [16]. The MMSE is a screening test for cognitive function that comprises 11 items and is widely used worldwide. Out of a maximum score of 30, a score less than 24 indicates dementia [17], and a score less than 28 indicates mild cognitive impairment (MCI) [18].

### 2.4. Physical Function

Physical function was assessed using grip strength, quadriceps muscle strength, one-leg standing time, 30 s chair stand (CS-30) test, timed up-and-go (TUG) test, and gait speed.

Grip strength was measured using a digital grip strength meter (T.K.K. 5401; Takei Kiki Kogyo Co., Niigata, Japan). The measurement method was based on a new physical fitness test in Japan [19]. The gripper width was adjusted so that the second joint of the index finger was close to the right angle. The measured limb was in the standing position, and participants were instructed to grasp the grip strength meter with maximum force. The participants were told that the digital grip strength meter should not come into contact with the body during the measurement [20]. Measurements were performed twice on each side, and the average of the maximum values on the left and right sides was used as the representative value.

Quadriceps muscle strength was measured using a muscle force measuring table for a single leg (T.K.K. 5715, Takei Kiki Kogyo Co., Niigata, Japan) and a tension meter (T.K.K. 5710 (e), Takei Kiki Kogyo Co., Niigata, Japan). Measurements were performed with reference to a previous study by Narazaki et al. [21]. The participants sat on the muscle force measuring table, with the hip and knee joints flexed at 90°. The participant’s distal lower legs were secured to a tension meter using a leg belt. From this position, the participants were instructed to extend their knee joints with maximal effort. Measurements were performed twice on each side, and the average of the maximum values on the left and right sides was used as the representative value.

The one-leg standing time was measured with reference to the new physical fitness test in Japan [19]. The time taken to hold the one-leg standing position with eyes open was measured using a digital stopwatch. During the measurement, participants were instructed to place both hands on their hips and gaze at a landmark 2 m away. The measurement was terminated when the lifted foot touched the floor or the supporting foot shifted position [22]. Measurements were performed twice on each side, with 120 s as the upper limit. The average of the maximum values on the left and right sides was considered the representative value.

The CS-30 test was performed using a chair that was approximately 40 cm high. The measurement method was based on a previous study by Jones et al. [23]. The starting position was arms crossed in front of the chest, and the participants were instructed to stand up as quickly as possible from that position and then sit down again [24]. The total number of repetitions in 30 s was recorded. If the movement was in progress at the end of 30 s, it was excluded from the total number of repetitions. The number of measurements was set to one.

The TUG test was measured using a chair approximately 40 cm high. The measurement method was based on a previous study by Wada et al. [25]. The starting limb position was the sitting position on a chair, and the measurement was started when the buttocks left the chair. The time taken to move around a cone set up 3 m in front of the participants and sit down again was measured using a digital stopwatch [26]. Participants were instructed to perform a series of actions with maximal effort. The number of measurements was one.

Gait speed was measured using a sheet-type foot pressure ground footprint measuring device (WalkWay MW-1000; Anima Co., Tokyo, Japan). This device measures the time and distance factors for walking under lower-limb loading. Participants walked barefoot on a 6.4 m walking path comprising a 2 m acceleration section, a 2.4 m measurement section, and a 2 m deceleration section [27]. The normal and maximum gait speeds were measured once each. Participants were instructed to “walk as fast as usual”, and when measuring the fastest gait speed, they were instructed to “walk as fast as possible”.

### 2.5. Body Composition

Body composition was measured using InBody 470 (InBody Japan Inc., Tokyo, Japan) [28]. InBody 470 is a body component analyzer based on the bioelectrical impedance method. Body weight, body mass index, body fat mass, and skeletal muscle mass were used as indicators.

### 2.6. Statistical Analysis

All variables were checked for normality using the Shapiro–Wilk test. Variables that followed normal distribution are shown as mean ± standard deviation. Variables that did not follow normal distribution are presented as medians (interquartile range). An unpaired *t*-test was used to compare the two groups of variables with normal distribution. Variables that did not follow normality were compared using the Mann–Whitney U-test. Cohen’s d or r was used as an indicator of effect size in the comparison between the two groups. The effect size was determined as Cohen’s d: small (d < 0.2), medium (d < 0.5), large (d < 0.8); r: small (d < 0.1), medium (d < 0.3), large (d < 0.5) [29]. First, for all participants, basic information and physical function were compared between two groups: one with strong subjective grip strength and the other with weak subjective grip strength. Next, comparisons were made separately between older adults with MMSE scores between 24 and 27 points, who fell into the MCI category [18], and those with scores between 28 and 30 points, who had normal cognitive function. Basic information and physical function were compared between the strong and weak subjective grip strength groups. IBM SPSS Statistics (version 29.0) was used for all statistical analyses, and the significance level was 5%.

## 3. Results

In all participants (*n* = 285), a comparison between the strong and weak subjective grip strength groups showed significant differences in terms of grip strength (*p* < 0.01), CS-30 test (*p* < 0.05), TUG test (*p* < 0.05), maximum gait speed (*p* < 0.05), and skeletal muscle mass (*p* < 0.01) (Table 1). 

Next, the strong and weak subjective grip strength groups were compared based on the participants’ cognitive function (Table 2). In the comparison between the two groups of cognitively normal older adults (*n* = 197), significant differences were found in grip strength (*p* < 0.01), quadriceps muscle strength (*p* < 0.01), one-leg standing time (*p* < 0.05), CS-30 test (*p* < 0.01), TUG test (*p* < 0.05), normal gait speed (*p* < 0.01), maximum gait speed (*p* < 0.01), and skeletal muscle mass (*p* < 0.01). Patients with low subjective grip strength had lower physical function and skeletal muscle mass. 

In contrast, no items showed significant differences between the two groups in older adults with MCI (*n* = 88) (Table 3).

## 4. Discussion

This study examined the relationship between subjective grip strength and physical function in older adults based on their cognitive function. The results showed that older adults with normal cognitive function showed significant differences in all measured physical functions and skeletal muscle mass between the two subjective grip strength groups. Patients with low subjective grip strength had lower physical function and skeletal muscle mass. In contrast, among older adults with MCI, there were no significant differences between the strong and weak subjective grip strength groups. The results indicated that subjective grip strength is an indicator of an overall decline in physical function and a decrease in skeletal muscle mass. However, this study indicated the need to consider cognitive function when assessing subjective grip strength in older adults. 

Comparisons between the two subjective grip strength groups revealed significant differences in grip strength, CS-30 test, TUG test, maximal gait speed, and skeletal muscle mass between groups. Patients with weak subjective grip strength had low physical function and skeletal muscle mass. Actual grip strength is not only an indicator of muscle strength but is also associated with various physical functions in older adults [30,31,32,33]. This means that grip strength is an indicator of physical function in older adults. Because actual grip strength is an index that reflects physical function, we assumed that subjective grip strength would also show a relationship with physical function. Older adults with weak subjective grip strength also had low skeletal muscle mass. Previous studies have reported that grip strength is associated with skeletal muscle mass [28,34]. This indicates that subjective grip strength is an index related to skeletal muscle mass as well as actual grip strength. Furthermore, these results indicate that subjective grip strength may exhibit similar characteristics to measured grip strength.

A comparison between the two subjective grip strength groups among older adults with MCI revealed no significant differences in terms of physical function. In contrast, the two subjective grip strength groups, including older adults with normal cognitive function, showed significant differences in all measured physical functions and skeletal muscle mass. Older adults with MCI have been reported to dissociate their actual performance from self-assessment [14,15]. It has also been reported that older adults with MCI overestimate their own abilities [35,36]. These findings suggest that even for physical functions that are simple to measure, such as grip strength, older adults with MCI may not be able to correctly recognize their own abilities. Therefore, we can infer that subjective grip strength did not reflect actual physical function in older adults with MCI, and no items showed significant differences between the two groups. These results indicate the need to consider participants’ cognitive function when assessing subjective grip strength in older adults. 

Older adults with normal cognitive function and weak subjective grip strength showed poor performance in multiple physical functions, such as muscle strength, balance, and gait ability. This indicates that subjective grip strength captures the overall decrease in physical function in older adults with normal cognitive function. The grip strength for older adults with weak subjective grip strength was 20.2 ± 3.9 kg, and the normal gait speed was 127.5 ± 24.7 cm/s. These measurements are above the diagnostic criteria for frailty [37] and sarcopenia [38] and are considered to indicate relatively well-preserved physical function. By identifying older adults with poor physical function in this population, interventions can be made before they exhibit an obvious functional decline. Thus, subjective grip strength may be a useful screening method for determining the need for preventive interventions.

The results of this study indicate the need to consider cognitive function when assessing subjective grip strength in older adults. Even when the degree of cognitive impairment is mild, there may be a discrepancy between perceptions and motor ability, suggesting that subjective measures do not reflect actual functioning. In contrast, older adults with normal cognitive function showed an association between subjective grip strength and actual physical function. Subjective grip strength has clinical value because it provides a simple assessment of physical function in situations where measurements are difficult. It is expected to be used in large-scale and non-face-to-face health surveys, as it can screen older adults with a high need for preventive interventions.

This study has several limitations. The study involved only older female adults who voluntarily participated in physical fitness sessions. Therefore, it is possible that this group was highly concerned about their own health, and their subjective indicators were more likely to reflect actual function. Moreover, because this was a cross-sectional study, we could not address the causal relationship between subjective grip strength and physical function. In the future, it is necessary to examine longitudinal changes by including older male adults and those who do not participate in physical fitness sessions. This is expected to increase the generalizability of the study results and clarify causal relationships, thereby demonstrating further applications of subjective grip strength.

## 5. Conclusions

This study examined the relationship between subjective grip strength and physical function in older adults based on cognitive function. The results indicate the need to consider cognitive function when assessing subjective grip strength in older adults. In older adults with normal cognitive function, weak subjective grip strength is an indicator of overall decline in physical function and loss of skeletal muscle mass. These results suggest that subjective grip strength can be used as a screening method to determine the need for preventive interventions that aim to preserve or improve physical function by capturing the physical function and skeletal muscle mass of older adults in situations where it is difficult to measure physical function.

## Figures and Tables

**Table 1:** Comparison between the two subjective grip strength groups.

	All Participants			
	Strong Group (*n* = 112)	Weak Group (*n* = 173)	*p*-Value	Effect Size
Age (years)	76.0 (72.3–81.0)	76.0 (72.0–81.0)	0.772	0.017
Height (kg)	51.1 (45.0–56.2)	48.6 (43.5–55.2)	0.228	0.071
BMI (kg/m^2^)	22.1 (20.3–24.2)	22.1 (19.7–24.0)	0.707	0.022
MMSE (points)	29.0 (27.0–30.0)	29.0 (27.0–30.0)	0.891	0.008
Grip strength (kgf)	22.3 ± 4.1	19.8 ± 4.2	<0.001 ^†^	0.598
Quadriceps muscle strength (kgf)	22.3 (17.0–28.1)	20.1 (16.2–24.8)	0.056	0.113
One-leg standing time (s)	15.3 (5.5–36.4)	12.5 (5.3–28.0)	0.267	0.066
CS-30 test (times)	22.0 (17.3–27.0)	20.0 (17.0–24.0)	0.039	0.122
TUG test (s)	5.8 (5.3–6.8)	6.2 (5.4–7.0)	0.027	0.131
Normal gait speed (cm/s)	133.7 (113.6–147.3)	126.3 (110.9–144.3)	0.071	0.107
Maximum gait speed (cm/s)	179.4 (151.6–199.9)	168.4 (150.4–188.6)	0.029	0.128
Body fat (kg)	15.5 (12.3–19.3)	15.7 (12.4–20.4)	0.429	0.047
Skeletal muscle mass (kg)	18.5 (16.7–20.0)	17.2 (16.1–18.8)	<0.001	0.213

Values are presented as mean ± standard deviation or median (interquartile range); Effect size unpaired *t*-test: Cohen’s d, Mann–Whitney U test: r; Strong Group: strong subjective grip strength group, Weak Group: weak subjective grip strength group; ^†^: unpaired *t*-test; for other items, the Mann–Whitney U test was used; BMI: body mass index, MMSE: Mini-Mental State Examination; CS-30: 30 s chair–stand test, TUG: timed up-and-go test.

**Table 2:** Comparison between the two subjective grip strength groups, including older adults with normal cognitive function.

	Normal Cognitive Function			
	Strong Group (*n* = 74)	Weak Group (*n* = 123)	*p*-Value	Effect Size
Age (years)	75.6 ± 5.8	76.2 ± 5.8	0.486 ^†^	0.103
Height (kg)	51.9 (45.7–56.6)	48.8 (43.5–55.5)	0.136	0.106
BMI (kg/m^2^)	22.2 (20.0–24.5)	21.9 (19.4–23.9)	0.338	0.068
MMSE (points)	30.0 (29.0–30.0)	30.0 (29.0–30.0)	0.201	0.091
Grip strength (kgf)	23.0 ± 3.7	20.2 ± 3.9	<0.001 ^†^	0.729
Quadriceps muscle strength (kgf)	23.2 (19.8–28.9)	20.1 (16.1–24.7)	0.009	0.244
One-leg standing time (s)	21.3 (6.9–45.5)	14.0 (6.5–29.9)	0.041	0.146
CS-30 (times)	23.0 (19.0–29.0)	20.0 (17.0–25.0)	0.002	0.220
TUG (s)	5.7 (5.2–6.4)	6.1 (5.3–6.8)	0.014	0.175
Normal gait speed (cm/s)	136.2 ± 18.7	127.5 ± 24.7	0.006 ^†^	0.383
Maximum gait speed (cm/s)	184.6 (164.9–204.0)	173.8 (152.3–191.4)	0.006	0.197
Body fat (kg)	16.1 ± 4.9	16.3 ± 6.3	0.833	0.031
Skeletal muscle mass (kg)	18.7 ± 2.3	17.7 ± 2.3	0.003 ^†^	0.447

Values are presented as mean ± standard deviation or median (interquartile range); Effect size unpaired *t*-test: Cohen’s d, Mann–Whitney U test: r; Strong Group: strong subjective grip strength group, Weak Group: weak subjective grip strength group; ^†^: unpaired *t*-test; for other items, the Mann–Whitney U test was used; BMI: body mass index, MMSE: Mini-Mental State Examination; CS-30: 30 s chair–stand test, TUG: timed up-and-go test.

**Table 3:** Comparison between the two subjective grip strength groups, including older adults with MCI.

	MCI			
	Strong Group (*n* = 38)	Weak Group (*n* = 50)	*p*-Value	Effect Size
Age (years)	79.2 ± 6.3	77.8 ± 5.9	0.291 ^†^	0.229
Height (kg)	48.9 (42.7–55.2)	48.6 (43.8–54.1)	0.930	0.009
BMI (kg/m^2^)	21.9 (20.5–24.0)	22.3 (20.2–24.2)	0.443	0.082
MMSE (points)	26.0 (25.0–27.0)	26.0 (25.0–27.0)	0.692	0.042
Grip strength (kgf)	20.3 ± 4.6	18.7 ± 4.6	0.109 ^†^	0.349
Quadriceps muscle strength (kgf)	17.6 (13.1–25.5)	20.9 (16.0–26.0)	0.259	0.120
One-leg standing time (s)	6.5 (3.8–18.6)	7.4 (4.0–20.6)	0.667	0.046
CS-30 (times)	19.0 (16.0–23.3)	19.5 (17.0–23.3)	0.499	0.032
TUG (s)	6.4 (5.6–6.9)	6.6 (5.8–7.4)	0.391	0.072
Normal gait speed (cm/s)	119.8 ± 26.4	122.8 ± 21.7	0.570 ^†^	0.123
Maximum gait speed (cm/s)	162.9 (136.4–186.9)	159.2 (145.1–177.3)	0.943	0.008
Body fat (kg)	14.8 (11.6–19.2)	15.8 (13.4–20.0)	0.212	0.133
Skeletal muscle mass (kg)	18.0 ± 2.6	17.2 ± 3.2	0.271 ^†^	0.239

Values are presented as mean ± standard deviation or median (interquartile range); Effect size unpaired *t*-test: Cohen’s d, Mann–Whitney U test: r; Strong Group: strong subjective grip strength group, Weak Group: weak subjective grip strength group; ^†^: unpaired *t*-test; for other items, the Mann–Whitney U test was used; BMI: body mass index, MMSE: Mini-Mental State Examination; CS-30: 30 s chair–stand test, TUG: timed up-and-go test.

## Data Availability

The data presented in this study are available upon request from the corresponding author. The data are not publicly available because they contain information that may infringe on the privacy of the study participants.
